# Innovative In Situ Interfacial Co-Assembled Lignin/Chitosan Nanoparticles—Green Synthesis, Physicochemical Characterization, In Vitro Release, and Intermolecular Interactions

**DOI:** 10.3390/ijms26146883

**Published:** 2025-07-17

**Authors:** Zhani Yanev, Denitsa Georgieva, Silviya Hristova, Milena Tzanova, Denitsa Nicheva, Boika Andonova-Lilova, Tzvetelina Zagorcheva, Diyana Vladova, Neli Grozeva, Zvezdelina Yaneva

**Affiliations:** 1 Faculty of Industrial Technology, Technical University of Sofia, 1756 Sofia, Bulgaria; zhani@abv.bg; 2 Department of Pharmacology, Animal Physiology, Biochemistry and Chemistry, Faculty of Veterinary Medicine, Trakia University, 6000 Stara Zagora, Bulgaria; denitsa.georgieva@trakia-uni.bg (D.G.); silviya.hristova@trakia-uni.bg (S.H.); 3 Department of Biological Sciences, Faculty of Agriculture, Trakia University, 6000 Stara Zagora, Bulgaria; milena.tsanova@trakia-uni.bg (M.T.); neli.grozeva@trakia-uni.bg (N.G.); 4 Institute of Electrochemistry and Energy Systems “Acad. Evgeni Budevski”, Bulgarian Academy of Sciences, 1113 Sofia, Bulgaria; d.vladimirova@iees.bas.bg; 5 Agrobiotechnology Department, AgroBio Institute, 8 Dragan Tzankov Blvd., 1164 Sofia, Bulgaria; boika_andonova@hotmail.com (B.A.-L.); tzvetelina.zagorcheva@gmail.com (T.Z.); 6 Research & Development & Innovation Consortium, 1784 Sofia, Bulgaria; 7 Department of Veterinary Anatomy, Histology and Embryology, Faculty of Veterinary Medicine, Trakia University, 6000 Stara Zagora, Bulgaria; diyana.vladova@trakia-uni.bg

**Keywords:** lignin, chitosan, nanoparticles, synthesis, quercetin, TEM, FTIR, XRD, in vitro release

## Abstract

In the present study, novel conjugated lignin/chitosan nanoparticles (LCNPs) were synthesized by a first-time simple green methodology using interfacial co-assembly between both biopolymers. The physicochemical (ζ-potential, size, concentration of surface acidic/basic groups), structural (surface functional groups), and morphological characteristics of the blank and quercetin-encapsulated (Q-LCNPs) nanoparticles were analyzed by the Boehm method, Fourier-transform infrared spectroscopy (FTIR), transmission electron microscopy (TEM), and X-ray diffraction (XRD). The experimentally determined encapsulation capacity was satisfactory—95.75%. The in vitro quercetin release efficiency in acidic solution that simulated the gastric microenvironment was 21.9%, followed by 68.5% and 99.8% cumulative release efficiency in simulated intestinal media at pH 7.4 and 6.8, respectively. The satisfactory applicability of the Weibull and sigmoidal mathematical models towards the experimental in vitro release data was indicative of the remarkable roles of diffusion and relaxation mechanisms.

## 1. Introduction

The latest scientific research on the synthesis and design of micro-/nanoformulations based on natural polymers is directed towards improving their therapeutic properties and achieving synergy in view of their bioactivities. Compared to synthetic polymers, biopolymers are distinguished by a number of advantages, such as availability, renewability, environmental friendliness, low cost, physiological compatibility, biodegradability, and safety [1,2].

The modern scientific literature strongly emphasizes the use of naturally derived polymers—such as collagen, cellulose, starch, chitin, chitosan, alginate, gelatin, and lignin—as promising materials for the fabrication of nanocarriers. These biopolymers offer customizable physicochemical properties, structure, and size, along with enhanced biofunctionalities. Their inherent biocompatibility, biodegradability, and bioactivity make them ideal candidates for a wide range of applications, including food packaging, energy storage, cosmetics, thermal and light stabilization, reinforced materials, drug delivery systems, UV protection, hybrid nanocomposites, and as antioxidant and antibacterial agents [3,4,5,6]. In this respect, according to recent scientific studies, lignin-based micro-/nanoparticles provide opportunities for the value-added utilization of lignin [7,8,9]. Moreover, novel investigations highlight various aspects of lignin-based hydrogels and micro-/nanoformulations regarding their applicability as UV blockers, antibacterial agents, and drug delivery systems, in virtue of their satisfactory biocompatibility, biodegradability, low cytotoxicity, satisfactory antioxidant potential, and sufficient reactive groups allowing for chemical modifications [10,11,12,13]. Recent studies have shown that using conjugated biopolymer formulations can overcome several limitations of individual biopolymer materials—such as poor mechanical strength, structural instability after implantation or transplantation, hydrophobicity, and low moisture resistance. At the same time, these formulations maintain or even enhance the biological functionality of the materials and offer more precise control over the encapsulation and release of natural bioactive compounds or pharmaceutical agents [14,15,16]. The essence of the design of nanoformulations based on the naturally derived polymers lignin and chitosan was provoked by the anticipated improvement of the physicochemical characteristics, ameliorated encapsulation efficiency, and sustained in vitro release capacity [17,18].

Among the main critical concerns of novel synthesis methodologies for the design of nanocarrier formulations based on biopolymers is the application of toxic chemicals (tetrahydrofuran, acetone, methanol, etc.), which limits their applicability in biomedicine, the pharmaceutical industry, and food technology due to the manifestation of possible toxic side effects [11,19,20,21]. Another crucial issue is the involvement of complicated, indirect, and tedious chemical reactions and processes that require expensive equipment [22,23,24]. To address the limitations and challenges related to the necessity for innovative methods in nanocarrier synthesis—such as the use of inexpensive, eco-friendly solvents; fast and simple processes requiring minimal equipment; and straightforward techniques for modification and characterization—this study introduces, for the first time, a novel green approach for producing and characterizing functional lignin/chitosan nanocarriers. These nanocarriers feature tunable size, high drug encapsulation efficiency, and controlled in vitro release profiles, making them suitable for applications in biomedical sciences, pharmaceutical technology, and the food industry.

The specific aim of the present study was to synthesize conjugated lignin/chitosan nanoparticles (LCNPs) by a novel green fabrication methodology, and to explore their physicochemical characteristics, encapsulation efficiency, and in vitro release potential.

## 2. Results and Discussion

### 2.1. Potentiometric Titration

Potentiometric titration is a powerful and cost-effective method for quantifying the functional groups existing on different materials. The titration method could provide more detailed information and in-depth properties, such as the contents and acid/basic dissociation constants pK_a_/pK_b_ of the functional groups.

**Figure 1:**
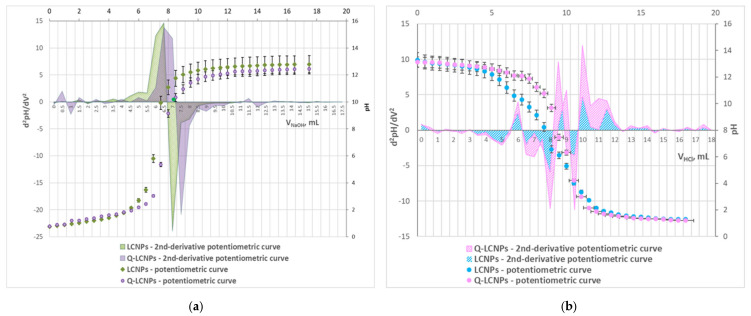
Zero-order and second-derivative potentiometric titration curves of LCNPs and Q-LCNPs: (**a**) titration of acidic sites; (**b**) titration of basic sites.

Figure 1 presents the zero-order and second-derivative potentiometric titration curves of LCNPs and Q-LCNPs. The plots allow for the determination of the equivalent volume of the titrant necessary to reach the equivalent point—the inflection point of the second-derivative plots. Based on this volume the calculated concentrations of the surface acidic functional groups of both types of NPs were 14 meq/g for LCNPs and 15 meq/g for Q-LCNPs. The stronger acidity of the surface of the flavonoid-loaded nanoparticles was also substantiated by the determined lower pK_a_ value (pK_a_ = 5.35) as compared to that for the unloaded nanoformulation—pK_a_ = 5.79. To calculate the basic functional groups of lignin/chitosan conjugated nanoparticles using potentiometric titration, the method involves determining the amount of protonated amine groups (–NH_3_^+^) in the chitosan structure, as chitosan is a polysaccharide derived from chitin, with amine groups that can be protonated. Based on the experimental data, the calculated concentrations of protonated amino groups were 18 meq/g for LCNPs and 18.7 meq/g for Q-LCNPs. The experimentally obtained pK_b_ values—6.57 and 7.46 for the unloaded and quercetin-encapsulated nanoparticles, respectively—are within the typical range of pK_b_ values of the amino group. The latter results prove the fact that, in more acidic conditions (pH < pK_a_), the amino groups predominantly exist in their protonated form (–NH_3_^+^).

The higher concentration of protonated surface amino groups on both types of nanoparticles, as compared to that of acidic groups, is indicative of the existence of intermolecular interactions between both polymers and the bioflavonoid.

### 2.2. ξ-Potential

ξ-Potential is a critical electrokinetic parameter that represents the electro-potential at the slipping plane of a dispersed particle relative to the bulk fluid media, determines the surface charge of drug delivery systems (e.g., liposomes, micro-/nanoparticles), and serves as a major factor in the assessment of colloidal suspension stability [25].

The experimentally determined values of the zeta potential of LCNPs and Q-LCNPs are presented in Table 1. The general boundary line between stable and unstable suspensions is assumed at ±30 mV. Particles with ξ-potential > +30 mV or <−30 mV are considered to be stable and strongly cationic and anionic, respectively [26]. The ξ-potential can influence the tendency of the nanoparticles to permeate cellular membranes. In this respect, cationic particles generally display higher toxicity associated with cell wall disruption, as most membranes are characterized with a negative charge [27]. Considering the above-mentioned assumptions, it could be concluded that both types of the conjugated lignin–chitosan NPs synthesized by us could be characterized as anionic, with the unloaded nanoformulation exhibiting a more pronounced anionic character. Additionally, both types of NPs could be classified as stable.

Similar results were reported by Sharma et al. (2023), who attributed the negative ξ-potential values of blank and meta-aminomethyl bisbenzylisoquinoline-loaded lignin nanoparticles to the presence of surface carboxylic, phenol, and hydroxyl functional groups [25,28,29].

### 2.3. UV/Vis Spectrophotometric Analyses

**Figure 2:**
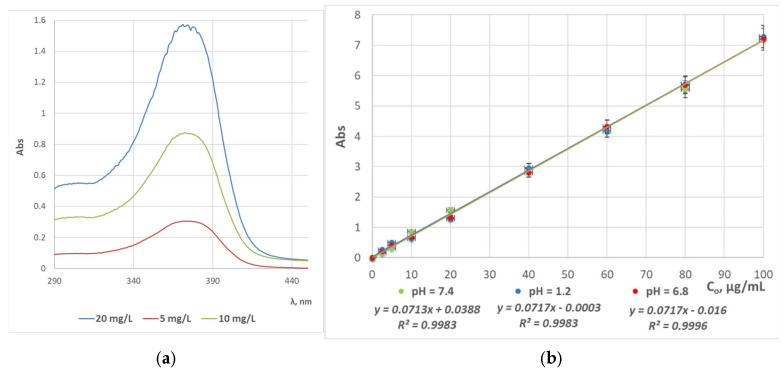
(**a**) UV/Vis spectra at pH = 7.4 and (**b**) standard calibration curves of quercetin in the three simulated microenvironments.

The concentrations of quercetin in EtOH were determined by UV/Vis spectrophotometry at a maximum absorption wavelength of λ = 372 nm. The corresponding UV/Vis spectra are presented in Figure 2a. The standard calibration curves of quercetin established at pH = 1.2, pH = 6.8, and pH = 7.4, so as to guarantee the accuracy of the experimental encapsulation and in vitro release results, are presented in Figure 2b. The three curves are characterized by very high linearity, approved by the high values of the regression coefficients (R > 0.9983).

### 2.4. TEM Analyses

**Figure 3:**
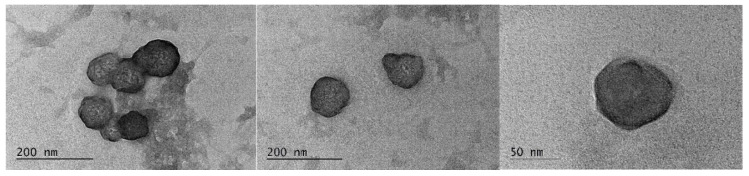
TEM images of LCNPs.

TEM images of the blank LNPs are depicted in Figure 3. The surface of the NPs appeared homogeneous, spherical, and regular. The photomicrographs displayed high density of the nanoformulations, which is indicative of the formation of polymeric networks between lignin and chitosan. The average size ranges presented in Table 1 for the blank and quercetin-loaded particles suggest an increase in size with the bioactive compound’s encapsulation. Similar results were reported for lignin–chitosan-based biocomposite films. According to Jassal et al. (2024), the biopolymer nature of the conjugated formulations is expected to provide improved oxygen supply to the skin surface and, consequently, better attachment to the epidermis layer [30]. The analysis of the observed aggregation of the flavonoid-loaded LCNPs (Figure 3) was especially challenging, and it was definitely attributed to the process of lyophilization, which led to the nanoparticles’ agglomeration.

### 2.5. XRD Analyses

X-ray diffraction analysis is among the most widely applied methods for determining the degree of crystallinity of polymer structures. The present study examined LCNPs and Q-LCNPs using X-ray powder diffraction analysis to determine their crystallinity and phase content.

The background was not subtracted from the diffractogram. The samples were placed in zero-background sample holders. Zero-background holders are made from 25.4 mm diameter silicon single crystal substrates. The silicon substrate is cut off so that it does not diffract during routine Bragg–Brentano geometry measurements using Cu radiation. One side of the substrate is flat, while the other side has a recess of 15 mm in diameter × 0.2 mm deep. Zero-background holders are used to mount small amounts of powder using dusting, smear, slurry, and similar techniques. They can also be used to support fibers, capillaries, and foil samples.

**Figure 4:**
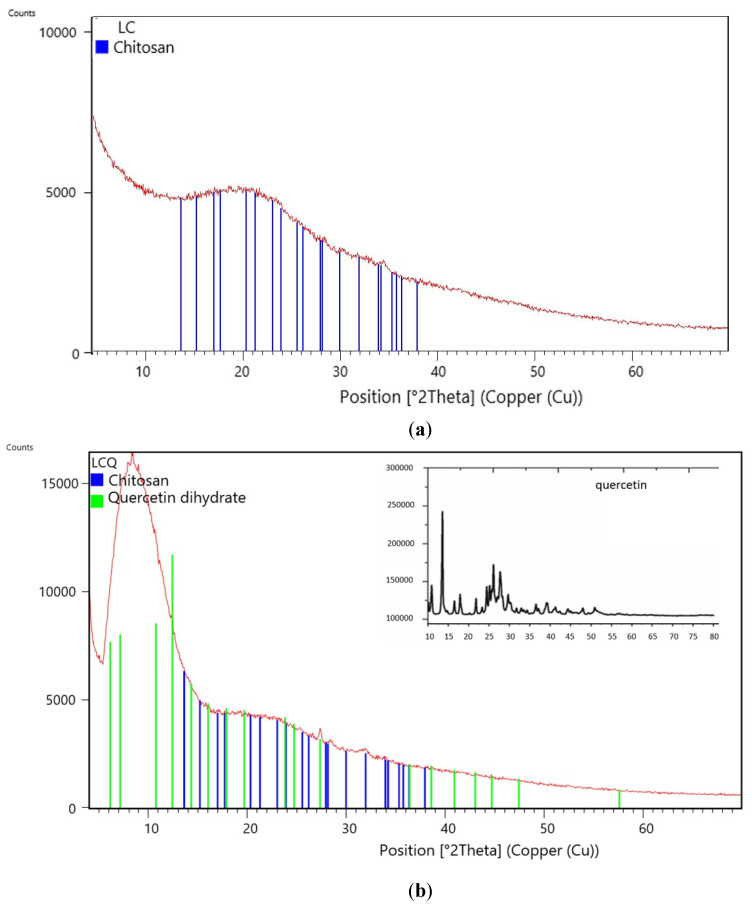
XRD diffractograms of (**a**) LCNPs and (**b**) Q-LCNPs and pure quercetin.

The diffractograms for both samples are presented in Figure 4a. Below the diffractograms, the positions of crystalline chitosan (PDF#00-039-1894) are shown with blue lines. Its strongest peaks, as well as the halo of LCNPs, are located in the range of 2θ = 21.6–21.8° [31]. The amorphous halo clearly indicates that the obtained lignin–chitosan particles are X-ray amorphous.

The diffraction pattern of Q-LCNPs (Figure 4b) shows two peaks above the background: at around 2θ = 8.00–9.00°, and again at around 2θ = 21.00°. In these regions, the most pronounced peaks of the compounds that we are looking for are found—chitosan (blue lines—PDF#00-039-1894) and quercetin dihydrate (green lines—PDF#00-038-1698).

Thus, the broad XRD peak could be attributed to the spectral characteristics of both biopolymers. However, the poor crystallinity characteristics are probably due to the fact that lignin comprises a three-dimensional polymer network of phenyl propane molecules, which is not a regular and ordered supramolecular structure [32].

The XRD spectrum of pure quercetin is characterized by various sharp peaks, reflecting its crystalline state (Figure 4b—green lines) [33]. The experimental data, however, displayed that most of the sharp crystal characteristic peaks of the bioflavonoid disappeared, except for the one at 2θ = 10°, providing convincing evidence of the encapsulation of this natural flavonoid within the structure of the biopolymer nanoparticles.

### 2.6. FTIR Analysis

**Figure 5:**
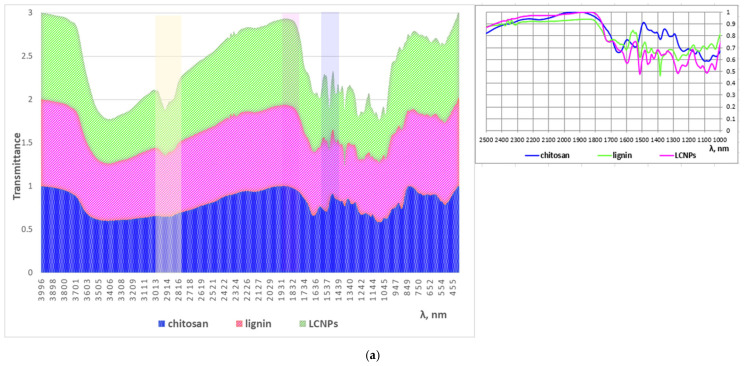

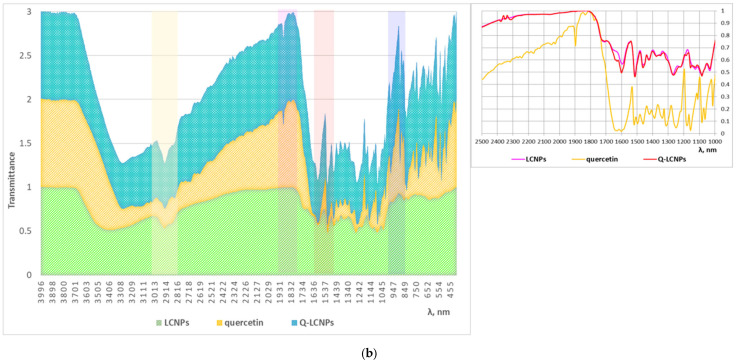
Comparative FTIR spectra of (**a**) pure chitosan, pure lignin, and LCNPs; and (**b**) LCNPs, pure quercetin, and Q-LCNPs.

FTIR analyses were applied to reveal the chemical structure of the novel conjugate lignin/chitosan nanoparticles on the bases of comparative estimation of the spectra of the pure precursors lignin and chitosan, the bioflavonoid quercetin, and the blank and quercetin-loaded NPs (Figure 5a,b, Table 2). The wide absorbance peaks of lignin, chitosan, and LCNPs at 3413, 3390, and 3200 cm^−1^ correspond to the total phenolic groups, N-H and O-H stretching vibrations, and intramolecular H bonds, while the sharper peaks at 3280 cm^−1^—characteristic of quercetin and, therefore, of the bioflavonoid-loaded formulations—correspond to O–H stretching band and dimeric hydroxyl O–H stretching. The well-defined peaks at 1549 and 1515 cm^−1^ on the LCNPs spectrum reflect the influence of the spectral characteristics of both polymers and are attributed to aromatic ring vibrations, C–H, O–H, and C=O bonds, and N-H bending of the primary amine, as well as to N-H bending of amide II. Moreover, in the LCNPs, the two characteristic bands of chitosan (amide I and amide II bands) are shifted from 1652 cm^−1^ to 1600 cm^−1^ and from 1550 cm^−1^ to 1515 cm^−1^, respectively, which is indicative of interaction of the hydroxyl groups in lignin with -OH and -NH_2_ groups in chitosan to form H bonds [34]. The strong effect of lignin’s polyphenol nature and the amino moieties in the chitosan macromolecules was proven by the high-intensity bands: at 1323 cm^−1^, assigned to the guaiacyl ring, methoxy C–O stretching, and O–H bonds of phenolic and non-ether groups; at 1263 and 1234 cm^−1^, attributed to syringyl units and C-N stretching of amide III; and at 1261 cm^−1^, characteristic of syringyl units on the LNPs spectrum (Figure 5a, Table 2).

The comparative estimation of the FTIR spectra of LCNPs, quercetin, and Q-LCNPs (Figure 4b) revealed that the peak at 3280 cm^−1^ corresponds to stretching absorption from associated hydroxyl groups in pure quercetin, while that at 1600 cm^−1^ corresponds to C=O stretching and C=C stretching, 1261 cm^−1^ to C-H stretching, and 1160-1130 cm^−1^ is derived from C-O-C stretching. Moreover, the spectrum of the loaded NPs revealed sharpening of the absorption band at 2906 cm^−1^, which was probably due to the great influence of C–H bond stretching in quercetin and to the C=O bond vibrations of –COOH, as well as C–H symmetric and asymmetric stretching vibrations of the LCNPs. The absorption bands of the loaded NPs within the wavelength range 1900–1850 cm^−1^ obviously carry the characteristic peaks of the natural flavonoid. In particular, the presence of the characteristic peaks of pure quercetin, and even their sharpening in the spectrum of Q-LCNPs, demonstrated that quercetin had been successfully embedded in the conjugated polyphenols framework.

### 2.7. Encapsulation Efficiency

The encapsulation efficiency (E, %) is a key parameter in the evaluation of the capacity of nanoparticles to incorporate biologically active compounds and drugs [38]. It is the percentage of the bioactive compound that is successfully encapsulated into the carrier. The encapsulation efficiency of the LCNPs was calculated based on the UV/Vis spectrophotometrically determined concentration of the flavonoid determined in the liquid phase after its encapsulation. The efficiency was established as 95.75% for a formulation synthesized by the application of an initial quantity of 20 mg of quercetin.

### 2.8. In Vitro Release Study

Simulating the in vitro release profile of quercetin from Q-LCNPs in physiologically relevant media, along with elucidation of the release mechanism, provides critical insights into the anticipated behavior of both the bioactive compound and the lignin/chitosan nanocarriers under in vivo physiological conditions. The latter would assist in the optimization of the design of various innovative pharmaceutical nanoformulations characterized by enhanced bioavailability. Beyond drug solubility, mass transfer through the biopolymeric matrix, matrix erosion potential, and the desorption capacity of the encapsulated bioactive compound, the physicochemical properties and structural characteristics of the nanocarrier system play a crucial role in determining the in vitro release kinetics of the loaded biomolecules [36]. Scientific studies report that the in vivo absorption and metabolism of quercetin or of quercetin glucosides take place predominantly in the small intestine [39]. The consecutive stages of the bioflavonoid pathway include phase II metabolism after the intestinal absorption, followed by final excretion into bile through the liver or urine through the kidneys [40].

**Figure 6:**
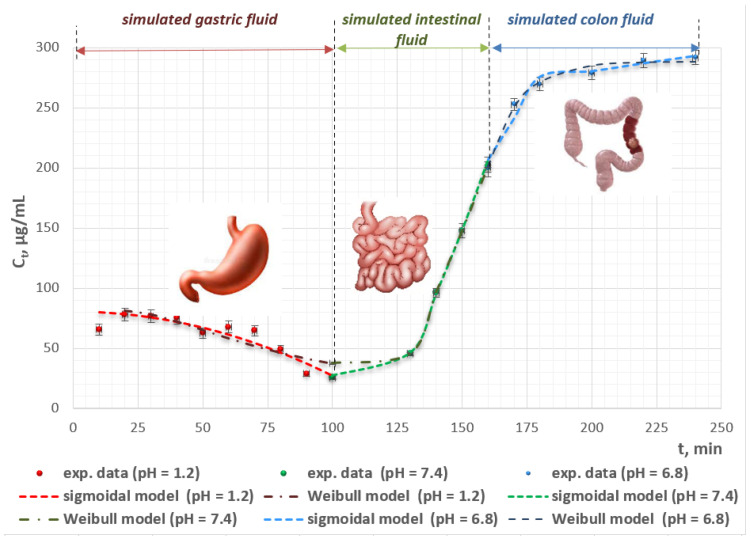
In vitro release profile of quercetin from LCNPs in simulated gastrointestinal media—experimental results; Weibull and sigmoidal model data.

The in vitro release behavior of quercetin from the Q-LCNPs in biorelevant simulated gastrointestinal media at pH = 1.2, 7.4, and 6.8 (mimicking the gastric, small intestinal, and colonic physiological compartments, respectively) was investigated. The experimental kinetic release curve, presented in Figure 6, exhibited triphasic release characteristics with an initial burst release, followed by a lag phase and, eventually, by slow and sustained release. The in vitro kinetics release curve comprises the following stages: (i) initial burst release of quercetin in gastric fluid (21.9% cumulative release efficiency); (ii) a steep, approximately vertical region displaying a higher release rate of the bioflavonoid in the small intestine within the time period 125–160 min (68.5% cumulative release efficiency); and (iii) a region of slow and sustained release up to 240 min in the colon (99.8% cumulative release efficiency). In the stomach, due to the strongly acidic medium, on the one hand, quercetin and lignin moieties exist predominantly in protonated form, which is an unfavorable factor concerning quercetin’s solubility and release from the hybrid nanocarrier through diffusion [41]. Scientific studies propose that acids serve as an antisolvent for alkaline lignin, which probably tightens the matrix around the incorporated biomolecules [36]. From another aspect, however, low pH supports chitosan’s solubilization, which consequently stimulates the erosion of the biopolymer network. The subsequent prolonged release corresponds to the liberation of quercetin through the dissolution, diffusion, or erosion of the conjugated matrix at neutral pH.

### 2.9. In Vitro Kinetics Modeling

Mathematical modeling was employed to analyze the kinetics of quercetin’s in vitro release from the synthesized lignin/chitosan nanoparticles by applying three mathematical models: the Korsmeyer–Peppas, Weibull, and sigmoidal models [42]. The suitability of the proposed models was assessed based on the values of the determined error functions. The values of the calculated model parameters are presented in Table 3. Obviously, based on the highest values of the regression coefficients and the lowest values of the SSE, MSE, and RMSE error functions, the Weibull and sigmoidal models were characterized by the best applicability towards the experimental data (Figure 5). The most indicative parameters in the Weibull model are the following: T, which denotes the lag time before the dissolution process begins; and the shape parameter b, which describes the kinetics release curve’s shape—exponential at b = 1, sigmoidal/S-shaped with upward curvature and an inflection point at b > 1, or parabolic with a steep initial slope followed by an exponential phase at b < 1. With b = −1.5 for release in gastric medium and b = 1 for the intestinal environment, the release behavior is indicative of a combination between exponential and parabolic release profiles, respectively.

The values of the diffusional constant k_s1_, the relaxation constant k_s2_, and the diffusional exponent n_s_ in the sigmoidal mathematical model enable the evaluation of the relative contributions of the mechanisms of relaxation and diffusion within the stages of the in vitro release process. The model data in Table 3 emphasize the significantly higher values of the kinetic constant k_s1_ for release in the stomach and in the small intestine as compared to k_s2_, which indicates the predominant role of diffusion over relaxation. On the other hand, in the colon, the relaxation mechanism of quercetin’s release dominated over diffusion. Obviously, the role of relaxation as a limiting mechanism of the bioflavonoid release from LCNPs is also significant. The latter could be attributed to the chemical nature of both biopolymers, which are susceptible to polymer chain relaxation caused by mechanical stress (agitation) and/or pH changes. The formation of compact supramolecular networks comprising lignin, chitosan, quercetin molecules, and water dipoles via intermolecular hydrogen bonding could also evoke sigmoidal behavior during the in vitro release process [43].

The Weibull and sigmoidal models have greater number of adjustable parameters and nonlinear terms that allow for better curve-fitting across the entire release profile. Moreover, both models are based on less restrictive assumptions, greater mathematical flexibility, and better applicability to the full time-course of release, not just the initial stages. They do not impose strict physical assumptions and are more descriptive rather than mechanistic. All of these facts make them better suited for complex release profiles where multiple transport mechanisms are at play. The Korsmeyer–Peppas model assumes diffusion-controlled release in polymer matrices. It is based on ideal conditions without swelling or erosion. Due to the complex contents of the newly fabricated nanoparticles, involving multi-mechanistic behavior, it fails to reliably describe the release behavior.

### 2.10. Intermolecular Biopolymer–Biopolymer Interactions in the Structure of LCNPs

**Scheme 1:**
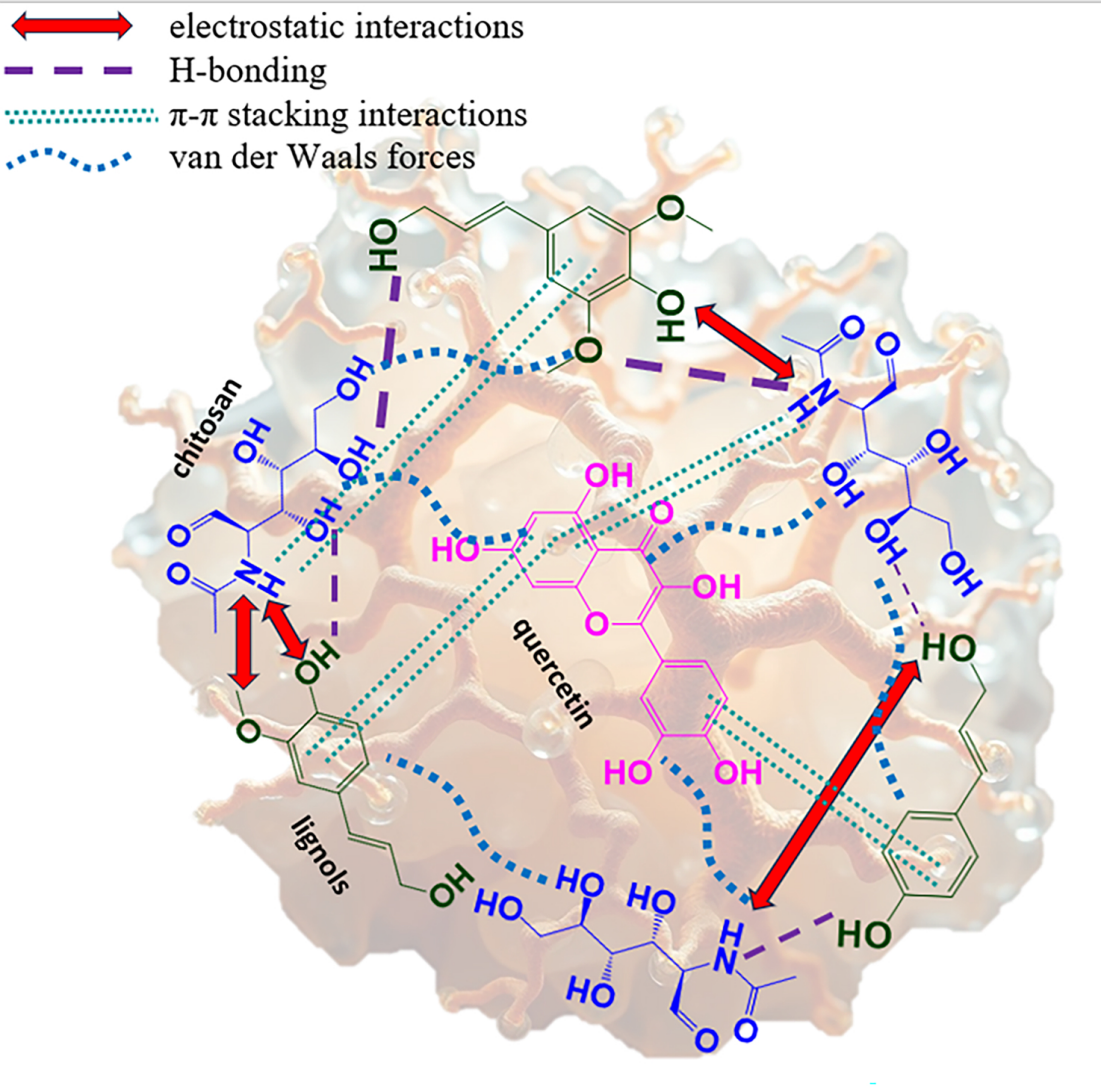
Probable intermolecular forces and interactions within the structure of the newly synthesized LCNPs and Q-LCNPs.

To obtain optimal properties of newly synthesized biopolymer nanoparticles and materials made thereof, it is crucial to control the interactions during both the particles’ production and their application [44]. Herein we focus on the current understanding of the probable interactions. Revealing the probable intermolecular interactions between the biopolymers lignin and chitosan is significant to analyze the behavior of the conjugated nanoparticles in vitro and in vivo (Scheme 1). Chitosan, as a cationic polymer due to the amino groups (-NH_2_), can form electrostatic interactions with the phenolic and carboxyl groups of alkaline lignin, which is negatively charged at certain pH values. The electrostatic forces are responsible for the stabilization of the composite structure and the enhancement of the mechanical properties of the conjugated polymer framework. Intermolecular H bonds can arise between the lignin -OH groups and the -NH_2_ or -OH groups of chitosan [45]. The aromatic backbone of lignin could participate in π-π stacking interactions with the -NH_2_ group of chitosan and the aromatic rings of quercetin molecules attached to chitosan macromolecules [40]. The H bonding and π-π stacking interactions probably enhance the structural integrity and stability of the LCNPs.

The physical and/or chemical crosslinking occurring between lignin and chitosan through interactions such as van der Waals forces or the entanglement of their molecular chains, as well as through reactive functional groups on both polymers, leads to the formation of complexes (or even precipitates) at favorable pH conditions. The pH of the solution plays a crucial role in the interactions between alkaline lignin and chitosan. At lower pH, chitosan is more protonated (positively charged) and solubilized, enhancing its ability to interact with the negatively charged groups of alkaline lignin. All of these types of interactions contribute to the stability and viscosity of the material, enhancing the mechanical and thermal properties of the composites [19].

## 3. Materials and Methods

### 3.1. Chemicals

The reagents applied in the present study—quercetin hydrate (C_15_H_10_O_7_·xH_2_O, CAS No: 849061-97-8), alkaline lignin (CAS No: 8068-05-1), chitosan (medium molecular weight, CAS No.: 9012-76-4), ethanol (EtOH, p.a. ≥ 99.8%), NaOH (p.a., HPLC), HCl (ACS reagent, 37%), and phosphate-buffered saline (PBS, P-3813)—were supplied by Sigma-Aldrich (St. Louis, MA, USA). Lactic acid (CH_3_CH(OH)COOH) (80%) was supplied by Chimtex, Bulgaria.

### 3.2. Synthesis of LCNPs

**Scheme 2:**
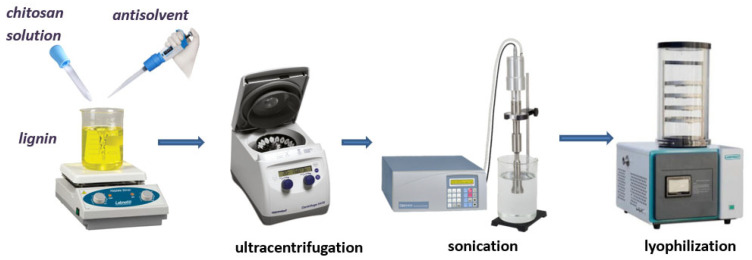
Synthesis methodology of LCNPs.

Blank LCNPs were synthesized by a combined method comprising solvent/antisolvent precipitation, self-assembly, sonication, and lyophilization (Scheme 2) [46]. To an aqueous solution of alkaline lignin with a concentration of 5000 mg/L containing 1 mL of 96% ethanol, we added 1 mL of 0.5% chitosan solution in 1% lactic acid at a flow rate of 0.5 mL/min. The resulting suspension was subjected to stirring on a magnetic stirrer for 30 min at 500 rpm, subsequent ultracentrifugation on a Hermle Z 326 K ultracentrifuge (HERMLE Labortechnik GmbH, Wehingen, Germany) at 15,000× *g* and temperature T = 10 °C, and washing of the nanoparticles with ultrapure water (INTEGRITY+ ultrapure water system, Adrona, Latvia). The third stage of the synthesis technique involved ultrasonic homogenization of the nanoparticle suspension on a Bandelin Sonopuls HD 2070 ultrasonic homogenizer (BANDELIN Electronic GmbH & Co. KG, Berlin, Germany) in an ice bath, at 93–96% intensity of ultrasound irradiation. The homogenized particles were subjected to subsequent lyophilization in a vacuum lyophilizer (Biobase Bioindustry Ltd., Jinan, China) at a temperature of T = −64 °C.

### 3.3. Encapsulation Study

The synthesis procedure of the Q-LCNPs followed the same steps, including the addition of 20 mg of the flavonoid dissolved in 96% ethyl alcohol to the aqueous solution of alkaline lignin prior to the addition of the chitosan solution in lactic acid. The concentration of the non-encapsulated flavonoid in the supernatant was determined spectrophotometrically on a DR 5000 UV/Vis spectrophotometer (Hach Lange, Düsseldorf, Germany) at the corresponding maximum wavelength of absorption. The encapsulation efficiency (EE, %) was determined by the following equation [42,47]:

(1) $$EE,\%=\frac{total\;quantity\;added\;compound-quantity\;of\;non-encapsulated\;compound}{total\;quantity\;added\;compound}\times 100$$

### 3.4. Surface Chemistry Characterization

The surface chemistry of LCNPs and Q-LCNPs was characterized by potentiometric titration, TEM, FTIR, XRD analyses, and ξ-potential.

The concentrations of acidic and basic surface sites on the LCNPs and Q-LCNPs were determined by the potentiometric titration methodology proposed by Boehm [48,49,50]. The total acidic sites were neutralized with 0.1 mol/L NaOH, and the basic sites with 0.1 mol/L HCl. The zero-order and second-derivative differential potentiometric titration curves were expressed as the pH value vs. the titrant volume (V, mL) and as d^2^pH/dV^2^ vs. V, mL, respectively.

The FTIR spectra of lignin, chitosan, quercetin, LCNPs, and Q-LCNPs were obtained with the potassium bromide (KBr) disc technique within the range 400–4000 cm^−1^, using a TENSOR 37 Bruker FTIR spectrometer (Bruker Optik GmbH, Ettlingen, Germany).

For the TEM analyses, the nanoparticle suspensions were stained with 1% uranyl acetate in 70% methanol and placed on electron microscopy membranes pre-coated with a thin layer of formvar membrane. TEM analyses were performed at high resolution using an HR STEM JEOL JEM 2100 transmission electron microscope (JEOL Ltd., Tokyo, Japan) operating at 200 kV, equipped with a GATAN Orius 832 SC1000 CCD camera (Gatan GmbH, Munich, Germany).

The structure of the nanoparticles, prepared by back-pressing the powder to effectively avoid the preferential alignment, was characterized using an X-ray diffraction (XRD) instrument (PANalytical Empyrean CuKα = 0.15406 nm, 40 kV, 40 mA) in a 2θ range from 4° to 70°.

The ξ-potential and particles sizes of the LCNPs and Q-LCNPs were measured using a Malvern particle analyzer at 25 °C. Each measurement was repeated in triplicate to check the reproducibility of the results.

### 3.5. In Vitro Release Studies

Experiments investigating the kinetics of the in vitro release of the encapsulated quercetin were carried out in a glass batch reactor equipped with a mechanical stirrer. The Q-LCNPs were stirred in enzyme-free simulated gastrointestinal physiological media at pH = 1.2, pH = 7.4, and pH = 6.8, at a temperature of T = 37 ± 0.5 °C, in a WNB 22 digital water bath (Memmert GmbH, Germany). Samples were taken at predetermined time intervals, and the quercetin concentrations in the simulated compartments were determined spectrophotometrically at λ = 390 nm. Volume corrections in the processing of the experimental results were performed by replacing the quantity of the withdrawn sample with an equivalent volume of fresh medium. The aim was to avoid saturation of the remaining solution. All experiments were performed in triplicate, and the average values were taken to minimize random error. Quercetin-free blanks and replicates at each release point were used for all experimental runs.

### 3.6. Mathematical Modeling; Statistical and Error Function Analyses

Three mathematical release models (the Korsmeyer–Peppas, Weibull, and sigmoidal function models), through nonlinear regression analyses, were used to interpret the experimental release data. The data obtained were expressed as means ± standard deviation (±SD) from three repetitions. The applicability of the mathematical models was assessed on the basis of the mode of the experimental and model release kinetics curves, as well as the values of the correlation coefficients (R^2^) and error functions (SSE, MSE, RMSE). The modeling approach was accomplished using XLSTAT BASIC+ statistical software for Excel (Microsoft Corporation, Redmond, WA, USA). The statistical significance was determined by performing Student’s *t*-test as the post hoc test. A value of *p* < 0.05 was considered statistically significant.

## 4. Conclusions

The results of the present study provide evidence that utilizing LCNPs synthesized from bio-based natural precursors could enhance the functionality of the novel nanoformulation, leading to high efficiency of encapsulation of bioactive compounds. While this was a model system, with encapsulated quercetin as a biologically active compound, the in vitro release experiments in simulated physiological gastrointestinal microenvironments displayed promising results associated with the high potential of the newly designed nanoparticles to provide sustained in vitro release. The interactions between alkaline lignin and chitosan, primarily governed by electrostatic forces, H bonding, and potentially π-π stacking intermolecular forces, could be geared for the facile design of various functional materials with enhanced structural properties and functionalities.

The next stage of this study will comprise the evaluation of the cytotoxicity of the LCNPs and detailed analyses of their in vitro antioxidant/radical-scavenging potential and antimicrobial activity. The anticipated positive impact is associated with the expected achievement of potentially applicable and feasible innovative nanoformulations as an ingenious drug delivery template in biomedical sciences and/or nutraceutical/phytocompound carriers in food technology, with additive/synergistic bioactive efficiency.

## 5. Patents

Yaneva, Z., Ivanova, D., Nikolova, G., Karamalakova, Y., Rusenova, N., Georgieva, E., Petkova-Parlapanska, K. 2024, Nanoparticle Compositions, Applicant: Trakia University, Stara Zagora, Bulgaria, GB Patent Application No: GB2415240.7 [47].

## Figures and Tables

**Table 1:** Average size, ξ-potential, and pH of blank and quercetin-loaded lignin/chitosan nanoparticles and their suspensions.

Nanoparticles	Size, nm	ξ-Potential, mV	pH
LCNPs	56.3–72.8	−39.51	9.15
Q-LCNPs	61.5–85.7	−31.68	8.57

**Table 2:** Assignment of the characteristic infrared bands on the FTIR spectra of the pure precursors lignin, chitosan, quercetin, and the novel nanoparticles—LCNPs and Q-LCNPs [35,36,37].

Wavenumber, cm^−1^	3700–3200	2950–2800	1700–1600	1590–1470	1500–1350	1350–1200	1200–1100	1100–1000	900–600
Lignin	3390 Phenolic hydroxyl groups	2935 C–H bond stretching; C=O bond vibrations of -COOH		1595 Aromatic ring vibrations and C–H, O–H, C=O bonds	1479; 1384 C=C–C ring bonding; C–H bonds of -CH_3_ groups	1301 Guaiacyl ring and methoxy C–O stretching; O–H bonds of phenolic and non-ether groups 1234 Syringyl units	1166 Guaiacyl unit band 1160–1130 -OH bonds of secondary alcohols; condensed aromatic rings	1058 Band of guaiacyl unit; -OH bonds of primary alcohols	893 Guaiacyl unit band
Chitosan	3200 N-H and O-H stretching; intramolecular hydrogen bonds.	2921; 2877 C-H symmetric and asymmetric stretching	1652 C=O stretching of amide I	1589 N-H bending of the primary amine 1550 N-H bending of amide II	1423; 1375 -CH_2_ bending; -CH_3_ symmetrical deformations	1325 C-N stretching of amide III	1153 Asymmetric stretching of the C-O-C bridge	1066; 1028 C-O stretching	
LCNPs	3413 Phenolic hydroxyl groups; N-H and O-H stretching; intramolecular hydrogen bonds.	2925 C–H symmetric and asymmetric stretching; C=O bond vibrations of -COOH	1600 C=O stretching of amide I C=C stretching	1549 Aromatic ring vibrations and C–H, O–H, C=O bonds; N-H bending of the primary amine; 1515 N-H bending of amide II;	1384 C=C–C ring bonding; C–H bonds of -CH_3_ groups bending; -CH_3_ symmetrical deformations	1323 Guaiacyl ring and methoxy C–O stretching; O–H bonds of phenolic and non-ether groups 1263; 1234 Syringyl units; C-N stretching of amide III 1261 syringyl units	1166 Guaiacyl unit band; -OH bonds of secondary alcohols; condensed aromatic rings; asymmetric stretching of the C-O-C bridge	1028 Band of guaiacyl unit; -OH bonds of primary alcohols C-O stretching	
Quercetin	3280 O–H stretching band; dimeric hydroxyl O–H stretching	2910 C–H bond stretching	1615 -C=O stretching vibration; double-bond alkenyl C=C stretching	1533 Aromatic ring vibrations and C–H, O–H, C=O bonds	1471 C=C–C ring bonding; C–H bonds of -CH_3_ groups	1238 C-H stretching; -C−OH deformation vibrations; -C-O-C bending	1153 Asymmetric stretching of the C-O-C bridge	1082 C-O-C stretching; −C−OH stretching	889 C–H vibration of aromatic ring 553–528 Bending vibration of -OH alcoholic groups
Q-LCNPs	3280 O–H stretching; dimeric hydroxyl O–H stretching; N-H and O-H stretching; intramolecular hydrogen bonds. phenolic hydroxyl groups	2906 C–H bond stretching; C=O bond vibrations of -COOH; C–H symmetric and asymmetric stretching	1600 C=O stretching of amide I; -C=O stretching vibration; double-bond alkenyl C=C stretching	1533 Aromatic ring vibrations and C–H, O–H, C=O bonds; N-H bending of the primary amine; 1515 N-H bending of amide II;	1384 C=C–C ring bonding; C–H bonds of -CH_3_ groups bending; -CH_3_ symmetrical deformations	1323 Guaiacyl ring and methoxy C–O stretching; O–H bonds of phenolic and non-ether groups 1261 Syringyl units; C-H stretching; -C−OH deformation vibrations; -C-O-C bending	1191 Guaiacyl unit band; phenolic C–O stretching vibration 1160–1130 -OH bonds of secondary alcohols; condensed aromatic rings; asymmetric stretching of the C-O-C bridge	1082 Band of guaiacyl unit; -OH bonds of primary alcohols; C-O-C stretching; −C−OH stretching	889 C–H vibration of aromatic ring 553–528 bending vibration of -OH alcoholic groups

**Table 3:** In vitro release kinetics model parameters and error function values.

Model		Gastric Medium (pH = 1.2)	Small Intestinal Medium (pH = 7.4)	Colonic Medium (pH = 6.8)
Korsmeyer–Peppas $\frac{C_{t}}{C_{0}}=a.t^{n}$	Model parameters	a = 330.808 n = −0.439	a = 8.461 × 10^−15^ n = 7.467	a = 7.623 n = 0.673
	Error functions	R^2^ = 0.629 SSE = 1185.901 MSE = 169.414 RMSE = 13.016	R^2^ = 0.985 SSE = 79.788 MSE = 79.788 RMSE = 8.932	R^2^ = 0.704 SSE = 1712.195 MSE = 428.049 RMSE = 20.689
Weibull $C_{t}=C_{o}.\left[1-e^{-\frac{{\left(t-T\right)}^{b}}{a}}\right]$	Model parameters	b = −1.5 C_o_ = 81.809 T = 5.767 a = 0.001	b = 1 C_o_ = 8.97 × 10^6^ T = 121.092 a = 1754770.820	b = 1 C_o_ = 288.244 T = 145.210 a = 12.304
	Error functions	R^2^ = 0.830 SSE = 580.262 MSE = 96.710 RMSE = 9.834	R^2^ = 1.000 SSE = 0.342	R^2^ = 0.989 SSE = 61.188 MSE = 20.396 RMSE = 4.516
Sigmoidal $\frac{C_{t}}{C_{0}}=k_{s1}.{\left(t-1\right)}^{n_{s}}$+ $k_{s2}.{\left(t-1\right)}^{n_{s}}$	Model parameters	k_s1_ = 0.277 n_s1 =_ −0.001 k_s2_ = 0.024 n_s2_ = −0.001	k_s1_ = 0.855 n_s1_ = 0.010 k_s2_ = 0.006 n_s2_ = 0.010	k_s1_ = 0.173 n_s1_ = 0.811 k_s2_ = 0.310 n_s2_ = 0.682
	Error functions	R^2^ = 0.858 SSE = 0.005 MSE = 0.001 RMSE = 0.028	R^2^ = 1.000 SSE = 1.054 × 10^−5^	R^2^ = 0.919 SSE = 0.002

## Data Availability

The datasets presented in this article are not readily available. Requests to access the datasets should be directed to the corresponding author. The data presented in this study are available upon request from the corresponding author.
